# Isolation and Genomic Characterization of a Novel Japanese Strain of *Pseudomonas protegens* and an Evaluation of Its Biocontrol Potential

**DOI:** 10.1264/jsme2.ME25086

**Published:** 2026-05-29

**Authors:** Rohyanti Yuliana, Saki Ikeda, Yuichiro Ikagawa, Kosei Yamauchi, Kotaro Kuwahara, Nur Adliza Baharom, Haruhisa Suga, Masafumi Shimizu

**Affiliations:** 1 Laboratory of Plant Pathology, The United Graduate School of Agricultural Science, Gifu University, 1–1 Yanagido, Gifu-shi, Gifu 501–1193, Japan; 2 Laboratory of Plant Pathology, Graduate School of Natural Science and Technology, Gifu University, 1–1 Yanagido, Gifu-shi, Gifu 501–1193, Japan; 3 Food Microbiology Laboratory, The United Graduate School of Agricultural Science, Gifu University, 1–1 Yanagido, Gifu-shi, Gifu 501–1193, Japan; 4 Natural Products Chemistry Laboratory, Faculty of Applied Biological Sciences, Gifu University, 1–1 Yanagido, Gifu-shi, Gifu, 501–1193, Japan; 5 Institute for Glyco-core Research (iGCORE), Gifu University, 1–1 Yanagido, Gifu-shi, Gifu 501–1193, Japan

**Keywords:** *Pseudomonas protegens*, biocontrol, pathosystem-dependent effects, comparative genomics, Japan

## Abstract

*Pseudomonas protegens*, a member of the *P. fluorescens* complex, is a key biocontrol bacterium with well-documented potential to protect plants against diverse pathogens. Although *P. protegens* strains have been widely exami­ned globally, those originating from Japan have not been well described. In this study, we isolated and characterized a new *P. protegens* strain, GSF-73, from the rhizosphere of *Allium fistulosum* in Gifu, Japan, and assessed its performance against several major plant diseases. Draft genome sequencing produced a 7.13-Mbp assembly, and average nucleotide identity values of 97.81–98.26% with reference strains (Cab75, CHA0, and Pf-5) confirmed its species identity. A comparative genomic anal­ysis showed that GSF-73 possessed a larger genome than the reference strains, containing eight conserved biosynthetic gene clusters for antimicrobial compounds and an expanded set of strain-specific genes related to metabolism, regulation, mobilome functions, and secretion. GSF-73 exhibited broad-spectrum antagonistic activity *in vitro* against fungal, oomycete, and bacterial pathogens. In biocontrol assays, GSF-73 significantly suppressed spinach Fusarium wilt, cucumber anthracnose on detached cotyledons, tomato bacterial wilt, and cucumber downy mildew. In contrast, root and/or seed treatments enhanced *Pythium* root rot in spinach and anthracnose in pot-grown cucumbers, indicating pathosystem-dependent efficacy. Despite these contrasting outcomes, GSF-73 shows strong biocontrol potential and merits further study to elucidate the mechanisms underlying both beneficial and adverse effects for its optimized use as a locally adapted biocontrol agent for Japanese agriculture.

Plant diseases markedly reduce global crop productivity, posing major threats to sustainable agriculture and global food security (Savary *et al.*, 2019). Although chemical pesticides continue to be widely used in the management of crop diseases, excessive reliance has generated environmental and health concerns, as well as an increase in the occurrence of pesticide-resistant pathogens. These issues highlight the need for eco-friendly and more sustainable disease-management strategies (Popp *et al.*, 2013; Ayilara *et al.*, 2023; Ediagbonya *et al.*, 2025). Therefore, biological control agents (BCAs), particularly plant-associated bacteria, have emerged as promising tools for suppressing plant pathogens while supporting crop growth (Ayaz *et al.*, 2023).

The genus *Pseudomonas* is among the most extensively studied groups of bacteria used as BCAs against soil-borne pathogens. These bacteria are abundant in soil ecosystems, produce diverse antimicrobial metabolites, and effectively colonize the roots of many plant species (Haas and Défago, 2005). *Pseudomonas protegens*, a species within the *P. fluorescens* complex, has drawn notable research interest because of its strong ability to protect both monocots and dicots from destructive soil-borne pathogens (Takeuchi and Seo, 2025). Strain CHA0, originally isolated from tobacco roots, is the most well-studied biocontrol strain of *P. protegens*. It strongly suppresses several destructive diseases, including tobacco black root rot caused by *Thielaviopsis basicola* and wheat take-all caused by *Gaeumannomyces graminis* var. *tritici* (Voisard *et al.*, 1994). Pf-5 is another prominent *P. protegens* strain discovered in cotton roots (Vick *et al.*, 2021); it exhibits the strong control of diseases caused by soil-borne pathogens, such as *Rhizoctonia solani*, *Pythium ultimum*, and *Boeremia exigua* (Howell and Stipanovic, 1979; Quezada-D’Angelo *et al.*, 2024). *P. protegens* typically synthesizes potent antimicrobial metabolites, including 2,4-diacetylphloroglucinol (DAPG) and pyoluteorin (PLT), along with multiple extracellular enzymes (Takeuchi and Seo, 2025). Some strains also produce additional antimicrobials, including hydrogen cyanide (HCN), pyrrolnitrin, and phenazine (Ramette *et al.*, 2011; Flury *et al.*, 2017; Someya *et al.*, 2017). Collectively, these characteristics make *P. protegens* one of the most promising BCAs currently available (Pellicciaro *et al.*, 2021).

Despite extensive worldwide studies on *P. protegens*, strains of this species discovered in Japan remain poorly documented. Strain Cab57, isolated from the rhizosphere of shepherd’s purse in a field in Hokkaido, is one of the few Japanese *P. protegens* strains exami­ned for biocontrol activity against plant pathogens (Takeuchi *et al.*, 2014). Since the ecological and physiological traits of bacteria often vary among strains (Van Rossum *et al.*, 2020), further investigation of Japanese *P. protegens* isolates is essential for obtaining a more detailed understanding of their diversity and for developing BCAs that are locally adapted.

In the present study, we exami­ned a new *P. protegens* strain from a collection of fluorescent *Pseudomonas* isolates obtained from various field soils and plant rhizospheres. We then assessed its potential as a biocontrol agent against several important plant diseases.

## Materials and Methods

### Isolation of P. fluorescens

We collected more than 91 plant samples and 29 bulk soil samples from multiple sites in Gifu Prefecture and Tottori Prefecture, Japan. To isolate rhizosphere-associated fluorescent pseudomonads, loosely adhering soil was removed from plant roots, which were then suspended in sterile deionized water (SDW). To obtain soil-dwelling fluorescent pseudomonads, bulk soil samples were similarly suspended in SDW. Both suspensions were agitated on a rotary shaker for 20 min, serially diluted, and plated onto King’s B (KB) agar medium (King *et al.*, 1954). After an incubation at 30°C for 24 h, colonies exhibiting green-to-blue fluorescence under ultraviolet (UV) light were isolated and purified via quadrant streaking. Purified isolates were preserved in 20% glycerol at –‍80°C for long-term storage.

### 16S rRNA gene sequence anal­ysis

To clarify the taxonomic position of fluorescent bacterial isolates, we analyzed their 16S rRNA gene sequences. Genomic DNA was extracted from overnight cultures grown in KB broth at 30°C with agitation at 200 rpm, using the NucleoSpin Microbial DNA Extraction Kit (Macherey-Nagel, GmbH) according to the manufacturer’s instructions. The 16S rRNA gene was amplified with the universal primers 27f and 1492r (Lane, 1991) under the following PCR conditions: an initial denaturation at 94°C for 2 min; 25 cycles of 94°C for 30 s, 55°C for 1 min, and 68°C for 2 min, and a final extension at 68°C for 5 min. PCR products (approximately 45 μL) were purified by adding 4.5 μL of 3 M sodium acetate and 135 μL of absolute ethanol, followed by centrifugation at 16,200×*g* at 4°C for 10 min (ST 8FR; Thermo Scientific). After removing the supernatant, the pellet was washed with 200 μL of 70% ethanol and centrifuged again under the same conditions. The resulting DNA pellet was dried at 80°C for 3 min and resuspended in 20 μL of ddH_2_O. Sequencing reactions were conducted using the BigDye Terminator v3.1 Kit (Applied Biosystems) with the primers 27f, 357f, 517r, and 1492r (Frank *et al.*, 2008), and products were analyzed on an ABI PRISM 3100 Genetic Analyzer (Applied Biosystems). Thermal cycling conditions were 96°C for 1 min, followed by 25 cycles of 96°C for 10 s, 50°C for 5 s, and 60°C for 4 min (Nishioka *et al.*, 2016). Sequence similarity searches were performed using BLAST against the GenBank database, and phylogenetic trees were reconstructed in MEGA v11.0 using the distance-based neighbor-joining (NJ) method (Saitou and Nei, 1987; Tamura *et al.*, 2013).

### Draft genome sequencing of strain GSF-73

Genomic DNA (gDNA) was extracted from strain GSF-73 following the protocol of Taboadela-Hernanz *et al.* (2025). Sequencing was performed by loading samples onto a Flongle Flow Cell (FLO-FLG114) on the MinION Mk1C platform (Oxford Nanopore Technologies), operating at 230 Hz and 40°C for 24 h with a minimum quality score of 9, using MinKNOW version 24.06.8. Base calling was conducted with Guppy v6.5.7. Raw reads were trimmed using Filtlong v0.2.1, with reads shorter than 1,000 bp being removed. The draft genome was assembled using Trycycler v0.5.4, which integrated Flye v2.9.2-b1786, Minipolish v0.1.2, and Raven v1.8.1, and was then polished with Racon v1.5.1. The genomic characteristics of strain GSF-73 were compared with those of Japanese *P. protegens* strain Cab57 (Takeuchi *et al.*, 2014) and model biocontrol strains of *P. protegens*, CHA0 (Jousset *et al.*, 2014) and Pf-5 (Paulsen *et al.*, 2005), isolated in Switzerland and the USA, respectively, using genome sequences obtained from the NCBI GenBank database. To visualize the genome structure of strain GSF-73, a circular chromosomal map was generated using BV-BRC version 3.55.17. Genome annotation of the assembled contigs was performed using the DDBJ Fast Annotation and Submission Tool (DFAST) (Tanizawa *et al.*, 2016). Average nucleotide identity (ANI) and tetranucleotide frequency correlation coefficients (TETRA) were calculated to assess the taxonomic relatedness of GSF-73 to reference strains (Parks *et al.*, 2015; Richter *et al.*, 2016).

### Genome annotation and functional prediction

The functional annotation of GSF-73 was performed based on the sequence similarity of the predicted protein sequences. Coding sequences (CDSs) were predicted from the draft genome using DFAST with default parameters. Similarity searches were conducted using BLAST+ (Camacho *et al.*, 2009). Gene Ontology (GO) terms were assigned using PANNZER2 (Törönen *et al.*, 2018) following the Gene Ontology Consortium nomenclature, and‍ ‍KEGG Orthology (KO) numbers were assigned using BlastKOALA (Kanehisa *et al.*, 2016). COG functional categories were assigned by rpsblast (BLAST+) searches against the NCBI Conserved Domain Database (CDD) (Camacho *et al.*, 2009), followed by classification according to the COG system (Galperin *et al.*, 2021). Gene functions were summarized based on COG categories, and genes assigned to multiple categories were counted in each relevant category.

Biosynthetic gene clusters (BGCs) involved in secondary metabolite and antimicrobial compound production were identified using antiSMASH (Blin *et al.*, 2023). Genes within each predicted BGC were mapped to locus tags.

To systematically evaluate genomic features related to plant colonization and antimicrobial activity, a comprehensive gene checklist was constructed by integrating KO, COG, GO, and antiSMASH annotations based on locus tags. This checklist consolidated the gene name, predicted product, functional annotations, and BGC information for each gene. Functions of interest were categorized based on antimicrobial biosynthetic pathways and regulatory systems, and representative marker genes or gene clusters were defined for each category. The presence of each function in strain GSF-73 was confirmed based on concordance among marker gene names, KO numbers, and where necessary, COG, GO annotations, predicted products, and positional consistency within antiSMASH-predicted BGCs.

### Comparative genomic anal­ysis

To compare functional category distributions among *P. protegens* strains, COG assignments were performed on the predicted protein sequences of each strain using the same annotation procedure described above. The number of CDSs assigned to each COG category was counted and visualized on a single graph to enable interstrain comparisons.

To assess CDS sharing among strains, a similarity-based comparative genomic anal­ysis was conducted using predicted protein sequences from *P. protegens* strains GSF-73, Cab57, CHA0, and Pf-5. All-versus-all BLASTp searches were performed using BLAST+ (Altschul *et al.*, 1990), and sequence identifiers were prefixed with strain-specific labels to avoid conflicts. Reciprocal best hit (RBH) relationships were defined using an E-value threshold of 1×10^–5^, sequence identity ≥60%, and query coverage ≥70%. RBH-linked CDSs were grouped into strain-corresponding CDS clusters (pseudo-orthogroups) and the presence or absence of each cluster in each strain was exami­ned. Shared and strain-specific CDS clusters were visualized using Venn diagrams generated with Venny 2.1.

In parallel, an orthogroup-based comparative anal­ysis was performed using OrthoFinder (version 3.0.1b1) (Emms and Kelly, 2019) with all-versus-all similarity searches conducted using DIAMOND (version 2.1.9) (Buchfink *et al.*, 2021). Orthogroups shared among strains and those specific to individual strains were identified based on the OrthoFinder output and visualized using Venn diagrams. GSF-73–specific genes were defined as orthogroups present exclusively in strain GSF-73 and absent from the other strains. Corresponding locus tags were extracted and integrated with the gene annotation checklist to generate a curated list of GSF-73-specific genes.

### Pathogens

The following pathogens were used in this study: *Pythium aphanidermatum* strain GUEh20Tol (stock culture, Gifu University Culture Collection, Gifu University); *Fusarium oxysporum* f. sp. *spinaciae* strain GF960 (provided by the Gifu Prefectural Agricultural Technology Center); *Colletotrichum orbiculare* strain 104-T (MAFF240422); *Ralstonia pseudosolanacearum* strain VT0801 (stock culture, Laboratory of Plant Pathology, Gifu University); and *Pseudoperonospora cubensis*, isolated from a cucumber leaf showing downy mildew symptoms.

### Inoculum preparation

*P. protegens* strain GSF-73 was cultured on KB agar at 30°C for 24 h. Cells from fresh colonies were transferred into KB broth and incubated for an additional 24 h with shaking at 200 rpm. Bacterial cells were then harvested by centrifugation at 9,000×*g* for 10 min, washed twice with sterile 10 mM MgCl_2_·6H_2_O, and resuspended in the same solution to an OD_600_ of 0.5 or 0.1.

*P. aphanidermatum* strain GUEh20Tol was cultured on V8 agar medium (Caten and Jinks, 1968) at 25°C in the dark for 24 h. After the incubation, 7-mm mycelial discs were cut from the colony’s periphery using a cork borer. Ten discs were then transferred into a 9-cm glass Petri dish containing 30 mL of autoclaved pond water (a 2:1 mixture of deionized water and filtered pond water) and incubated at 25°C in the dark for 24 h. Zoospores were subsequently harvested and adjusted to 1×10^4^ zoospores mL^–1^ using a hemocytometer.

*F. oxysporum* f. sp. *spinaciae* strain GF960 was cultured on potato sucrose agar (PSA) at 25°C for 4 days in the dark. Mycelial discs taken from the colony edge were transferred to potato sucrose broth and incubated at 25°C for 3 days with shaking at 120 rpm. The resulting culture broth was filtered through three layers of sterile gauze to remove mycelial fragments and centrifuged at 5,000×*g* for 5 min. The pellet containing spores was resuspended in SDW, and the concentration was adjusted to 1×10^4^ spores mL^–1^.

*C. orbiculare* strain 104-T was grown on PSA at 25°C for 7 days. Spores produced on the colony surface were suspended in SDW and adjusted to 5×10^5^ spores mL^–1^ for the detached cotyledon assay and 1×10^5^ spores mL^–1^ for the detached leaf assay, using a hemocytometer.

*R. pseudosolanacearum* strain VT0801 was cultured on casamino acid–peptone–glucose agar (CPGA) medium (Kelman, 1954) at 30°C and incubated for 48 h. Cells from the colony were inoculated into CPG broth and cultured at 30°C for 24 h with shaking at 200 rpm. The cells were harvested by centrifuging the broth at 9,000×*g* for 10 min, resuspended in 10 mM MgCl_2_·6H_2_O, and adjusted to an OD_600_ of 1.0.

*P. cubensis* was routinely subcultured on detached cucumber leaves placed on moist Kimwipe tissues in a Petri dish. The dish was maintained at 20°C under a 12-h light/12-h dark cycle. An inoculum of *P. cubensis* was prepared from cucumber leaves exhibiting downy mildew symptoms. Five to ten leaf disks were cut from infected tissue, submerged in distilled water, and then gently agitated using a vortex mixer to release *P. cubensis* spores. The spore concentration was adjusted to 1×10^4^ spores mL^–1^ using a hemocytometer.

### Plant materials

Spinach (cv. Banchu-summer-sky), cucumber (cv. Tokiwa-jibai), and tomato (cv. Ponderosa) were used for experiments. Seeds were surface-sterilized by immersion in 70% ethanol for 1 min followed by 2% sodium hypochlorite for 3 min, and then rinsed six times with SDW. In the cucumber detached leaf assay, seeds were additionally soaked in a bacterial suspension (OD_600_=0.1) for 10 min. Sterilized seeds were placed on moistened filter paper in Petri dishes and incubated in the dark until germination. Incubation periods were at 20°C for 3 days for spinach, at 25°C for 3 days for cucumber, and at 30°C for 3 days for tomato.

### *In vitro* antagonism assay

This experiment evaluated the antagonistic activity of GSF-73 against fungal, oomycete, and bacterial pathogens. Activity against fungal and oomycete pathogens was assessed using the dual-culture method, while activity against the bacterial pathogen was exami­ned using the spot-on-the-lawn method (Li *et al.*, 2020) with minor modifications.

In the dual-culture assay, GSF-73 was pre-cultured on KB agar at 30°C for 24 h, after which a loopful of cells was inoculated 1.5 cm from the edge of fresh 9-cm PSA plates. The test pathogens were pre-cultured on PSA. Agar plugs (7 mm in diameter) were cut‍ ‍from the leading edges of 1-day-old colonies of *P. aphanidermatum* strain GUEh20To1, 7-day-old colonies of *C. orbiculare* strain 104-T, and 4-day-old colonies of *F. oxysporum* f. sp. *spinaciae* strain GF960. Each pathogen plug was placed 1.5 cm from the opposite side of the PSA plate relative to GSF-73 and incubated at 30°C, with incubation periods of 1 day for *P. aphanidermatum* and 10 days for the remaining pathogens. Regarding *C. orbiculare*, plates were pre-incubated for 7 days before inoculating GSF-73. Pathogens grown alone served as controls. The percentage inhibition of radial growth was calculated using the following formula: % inhibition=(*R_1_*–*R_2_*)/*R_1_*×100%, where *R_1_* and *R_2_* represent the colony radii of the pathogen in the control and GSF-73 treatments, respectively.

In the spot-on-the-lawn assay, *R. pseudosolanacearum* strain VT0801 was cultured on 9-cm CPGA plates (Kelman, 1954) at 30°C for 24 h. After the incubation, VT0801 cells were suspended in 2 mL of SDW, and 100 μL of the suspension was spread onto a fresh 9-cm PSA plate. A 7-mm agar plug taken from a 2-day-old King’s B culture of GSF-73 was placed at the center of the inoculated plate. Plates were incubated at 30°C for 2 days, and inhibition was evaluated by the presence of a clear halo surrounding the GSF-73 plug. All antagonism assays were conducted in three independent trials, each including three technical replicates per treatment (*n*=9).

### Biocontrol potential against bacterial wilt in tomato

The efficacy of GSF-73 in controlling tomato bacterial wilt was assessed using a seedling bioassay based on Tanaka *et al.* (2025) with slight modifications. Briefly, five pregerminated tomato seeds were sown in autoclaved vermiculite within a flat-bottomed glass tube (25×125 mm; AGC Techno Glass). The seeds were then drench-treated with a 2-mL aliquot of the GSF-73 suspension (OD_600_=0.5) and drench-inoculated with a 2-mL aliquot of the pathogen cell suspension (OD_600_=1.0). Seeds treated with a 10 mM MgCl_2_·6H_2_O solution instead of GSF-73 and subsequently challenged with the pathogen served as the mock control. The seeds were maintained in a controlled-environment chamber (BioTRON LH-241PFDT-SP; NK System) at 28°C under a 12-h light/12-h dark cycle. Three replicate tubes were used for each treatment, and the experiment was repeated three times. After a 7-days incubation, symptoms of bacterial wilt were scored on a disease scale from 0 to 3, where 0=no symptoms, 1=small necrotic areas on the hypocotyl, 2=a wilted seedling or extensive necrosis, and 3=dead or no germination, as described by Marian *et al.* (2018). The area under the disease progress curve (AUDPC) value of each plant was calculated as described above.

### Biocontrol potential against *Pythium* root rot in spinach

A double-autoclaved mixture of commercial potting soil (Nippi gardening soil No. 1; Nihon Hiryo) and vermiculite at a 20:1 ratio was placed into a 42-cell tray (Beepot Y-49; Canelon Kakou). Pregerminated spinach seeds were sown in the tray and maintained in a controlled environmental chamber at 25°C with a 12-h photoperiod for 7 days. The seedlings were drench-inoculated with 5 mL of the *P. aphanidermatum* zoospore suspension and 5 mL of the GSF-73 cell suspension (OD_600_=0.5). Seedlings drenched with 10 mM MgCl_2_·6H_2_O instead of the GSF-73 suspension served as mock controls. The inoculated seedlings were kept in the same chamber. There were 10 seedlings per treatment, and the experiment was performed three times. Seven days after the incubation, disease severity was rated on a scale of 0–3, where 0=no symptoms, 1=cotyledon leaves show slight wilting or yellowing, 2=cotyledon leaves and some true leaves show slight wilting or yellowing, and 3=the entire seedling shows severe wilting or dies. The AUDPC value for each plant was calculated using the following formula: AUDPC=Σ [0.5 (*x_i_*+*x_i–1_*)] (*t_i_*–*t_i–1_*), where *x_i_* and *x_i–1_* are disease severities at times *t_i_* and *t_i–1_*; *t_i_* and *t_i–1_* are consecutive evaluation dates; and *t_i_*–*t_i–1_* equals 1.

### Biocontrol potential against Fusarium wilt in spinach

The biocontrol efficacy of GSF-73 against Fusarium wilt caused by *F. oxysporum* was evaluated in spinach. Spinach seedlings were grown in a 42-cell tray for 7 days as described above. The seedlings were then drenched with 5-mL aliquots of a spore suspension of *F. oxysporum* f. sp. *spinaciae* and 5-mL aliquots of a GSF-73 cell suspension (OD_600_=0.5). As the mock control, seedlings were drenched with 10 mM MgCl_2_·6H_2_O instead of GSF-73. The inoculated seedlings were subsequently maintained under the same conditions. There were 10 seedlings per treatment, and the experiment was conducted three times. Seven days after the incubation, disease severity was evaluated on a scale of 0–3, where 0=no symptoms, 1=cotyledon leaves show slight wilting or yellowing, 2=cotyledon leaves and some true leaves show slight wilting or yellowing, and 3=the entire seedling shows severe wilting or dies. The AUDPC value of each plant was calculated as described above.

### Biocontrol potential against anthracnose in cucumbers

In the present study, we exami­ned the efficacy of GSF-73 against cucumber anthracnose using two independent approaches: a detached cotyledon assay and a pot experiment.

The detached cotyledon assay was conducted following Shimizu *et al.* (2009) with slight modifications. Briefly, pregerminated seeds were sown in double-autoclaved commercial soil (Saika Ichiban, Ibigawa Kogyo) and maintained in a controlled environmental chamber at 25°C with a 12-h light/12-h dark cycle for 5 days. Cotyledons were excised from the seedlings and immersed in a GSF-73 suspension (OD_600_=0.1) supplemented with 0.01% Silwet L-77 for 2 min. Cotyledons immersed in 10 mM MgCl_2_·6H_2_O containing 0.01% Silwet L-77 served as mock controls. The cotyledons were placed abaxial side down on 1.5% water agar in 9-cm Petri dishes (four cotyledons per plate) and incubated for 24 h under the same conditions. A 5-μL droplet of the *C. orbiculare* spore suspension was then applied to the cotyledons (two spots per cotyledon), and they were incubated for an additional 7 days for a disease assessment. The experiment was repeated three times.

A pot experiment was conducted as follows: pregerminated cucumber seeds were immersed in a GSF-73 cell suspension (OD_600_=0.5) for 10 min and then sown in 9-cm pots filled with 200 g of a double-autoclaved mixture of field soil and commercial soil mix (Nippi gardening soil No. 1) at a 1:1 ratio. The seeds were again treated with GSF-73 by drenching them with 8 mL of the cell suspension and subsequently fertilized with 30 mL of a 250-fold diluted Hyponex solution (type 6–9–5; Hyponex Japan). Fourteen days after the incubation in a controlled-environment chamber at 25°C with a 12-h light/12-h dark cycle, the seedlings were drenched with another 8 mL of the GSF-73 suspension. They were then maintained under the same growth conditions for an additional 7 days. Cucumbers treated with 10 mM sterile MgCl_2_·6H_2_O in place of the GSF-73 suspension served as mock controls. Twenty 20-μL drops of the *C. orbiculare* spore suspension supplemented with 0.01% Silwet L-77 were then applied to the surfaces of the first and second true leaves, and a lens-cleaning paper disk was placed over each drop. The inoculated plants were incubated in a humid chamber (KCLP-1400 ICTS; NK System) at 100% relative humidity and 23°C for 30 h. They were subsequently transferred to a controlled-environment chamber (25°C, 12-h light/12-h dark cycle) for 6 days for a disease assessment. There were 10 plants in each treatment, and the experiment was repeated six times.

At the end of the incubation period, the sizes of necrotic lesions on cotyledons and leaves were quantified using ImageJ software, following the procedure of Shimizu *et al.* (2009). The cotyledons, or leaves, were scanned with a desktop scanner, and the resulting images were saved in the JPEG format. Lesion areas were subsequently measured using ImageJ version 1.54 (National Institutes of Health, USA; https://imagej.net/nih-image/).

### Biocontrol potential against downy mildew in cucumber

The biocontrol efficacy of GSF-73 against downy mildew in cucumbers was evaluated as follows: Pregerminated cucumber seeds were sown in a 42-cell tray and maintained in a controlled environmental chamber at 20°C with a 12-h light/12-h dark cycle for 10 days. The seedlings were subsequently transplanted into 9-‍cm pots containing a double-autoclaved commercial soil mix (Saika Ichiban). Twenty-one days after transplanting, the plants were drenched with 50 mL of the GSF-73 suspension (OD_600_=0.5), while mock controls received an equal volume of 10 mM MgCl_2_·6H_2_O. The cucumber plants were then challenged with *P. cubensis* by thoroughly spraying a spore suspension supplemented with 0.01% (v/v) Silwet L-77 onto both abaxial and adaxial leaf surfaces. The inoculated plants were incubated in a humid chamber (KCLP-1400 ICTS) at 100% relative humidity at 20°C for 17 days. Disease severity was assessed using the scale described by Charoenwattana *et al.* (2017): 0=no infection; 1=1–5% leaf area infected; 2=6–25% leaf area infected; 3=26–50% leaf area infected; 4=51–75% leaf area infected; and 5=76–100% leaf area infected. Disease severity was calculated using the following formula: Disease severity=([number of diseased leaves in each disease scale×disease scale]/[total number of leaves evaluated×highest disease scale])×100%. Five plants were included in each treatment, and the experiment was performed three times.

### Statistical anal­ysis

All statistical anal­yses were performed with Prism 10.6.1 and R version 4.5.1.

### Nucleotide sequence accession numbers

The draft genome sequence of strain GSF-73 has been deposited in the DDBJ database under accession no. AP045182.

## Results

### Isolation of P. fluorescens

A total of 120 fluorescent bacteria were collected. A subsequent 16S rRNA gene sequence anal­ysis revealed that 85 strains belonged to the genus *Pseudomonas*, while the remaining 35 strains were classified into other genera (Table S1). Among the *Pseudomonas* strains, 35 belonged to the *P. fluorescens* complex. Within *P. fluorescens* complex strains, only strain GSF-73, isolated from the rhizosphere of Welsh onion (*Allium fistulosum*) collected in Sekigahara, Gifu, was the most closely related to *P. protegens*.

### Draft genome sequencing of GSF-73

**Fig. 1.**
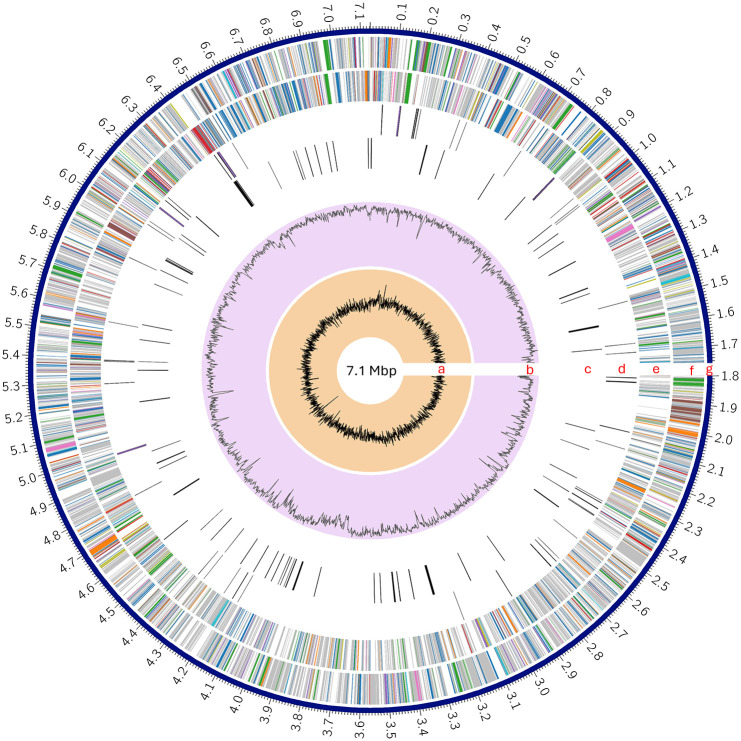
Chromosomal map of *Pseudomonas protegens* strain GSF-73. From the center outward, circle (a) shows GC skew, circle (b) indicates GC content (%), circle (c) highlights rRNA genes, circle (d) indicates tRNA genes, circle (e) represents protein-coding sequences (CDSs) on the forward strand, circle (f) represents CDSs on the reverse strand, and circle (g) shows the positions of predicted genes along the chromosome. The total genome size is approximately 7.13 Mb.

The resulting draft genome of GSF-73 had a total sequence length of 7,132,059 bp with a GC content of 63.0%, comprising 6,705 CDSs, 16 rRNA genes, 74 tRNA genes, and a coding ratio of 88.7% (Fig. 1). A circular chromosomal map revealed the overall genome organization, including the distribution of CDSs, rRNA and tRNA genes, GC content, and GC skew across the chromosome. A comparative anal­ysis of general genomic features among *P. protegens* strains GSF-73, Cab57, CHA0, and Pf-5 is summarized in Table 1. Among the four strains, GSF-73 possessed the largest genome, exceeding those of the well-characterized biocontrol strains Cab57 (6.83 Mb), CHA0 (6.87 Mb), and Pf-5 (7.07 Mb). Consistent with its larger genome size, GSF-73 also contained the highest number of predicted CDSs. In contrast, the G+C content and the numbers of rRNA and tRNA genes were highly similar among all strains exami­ned, indicating overall genomic conservation within the species.

An average nucleotide identity (ANI) anal­ysis showed that GSF-73 shared ANI values ranging from 97.81 to 98.26% with *P. protegens* strains, including Cab57, CHA0, and Pf-5 (Table 2), exceeding the established species delineation threshold of 95–96%. In addition, TETRA values between GSF-73 and the three reference strains were all >0.999, further supporting their close genomic relatedness. Based on these results, GSF-73 was classified as a distinct strain within the species *P. protegens*.

### Genome annotation and functional prediction

The antiSMASH anal­ysis revealed 19 putative BGCs for secondary metabolites in the GSF-73 genome (Table 3 and S2). These included BGCs corresponding to eight antimicrobial compounds previously reported in *P. protegens*—protegencin, orfamides, HCN, PLT, enantio-pyochelin, pyrrolnitrin, pyoverdine, and DAPG. Consistent with these predictions, marker genes and gene clusters associated with antimicrobial biosynthesis were detected, including protegencin (antiSMASH region001), orfamide A/C (*ofaA–C*; region006), the HCN production system (*hcnA–C*), pyoluteorin (*plt*), pyrrolnitrin (*prnA–D*), and DAPG (*phlA–D*, *phlG*) biosynthetic clusters (Table 4). In addition, genes involved in siderophore biosynthesis and uptake, including those for enantio-pyochelin (*pchA–C*, *pchE*, *pchF*, and *pchR*) and pyoverdine (*pvdA*, *pvdE*, and *fpvA*), as well as components of the global Gac/Rsm regulatory system, were conserved.

In addition to BGCs corresponding to all major antimicrobial metabolites previously reported for *P. protegens*, an antiSMASH anal­ysis identified several NRPS-, RiPP-like-, β-lactone-, and CDPS-type gene clusters that did not show confident similarity to any characterized BGCs (Table 3 and S2). Therefore, these clusters were classified as uncharacterized secondary metabolite gene clusters.

COG-based functional annotation assigned 15,621 functional category entries to genes in strain GSF-73 (Fig. S1). The most abundant categories were [E] amino acid transport and metabolism, [T] signal transduction mechanisms, [R] general function prediction only, [K] transcription, and [P] inorganic ion transport and metabolism, together accounting for 48.3% of all assignments. The top 10 COG categories collectively represented 74.5% of the total functional assignments.

### Comparative genomic anal­ysis

**Fig. 2.**
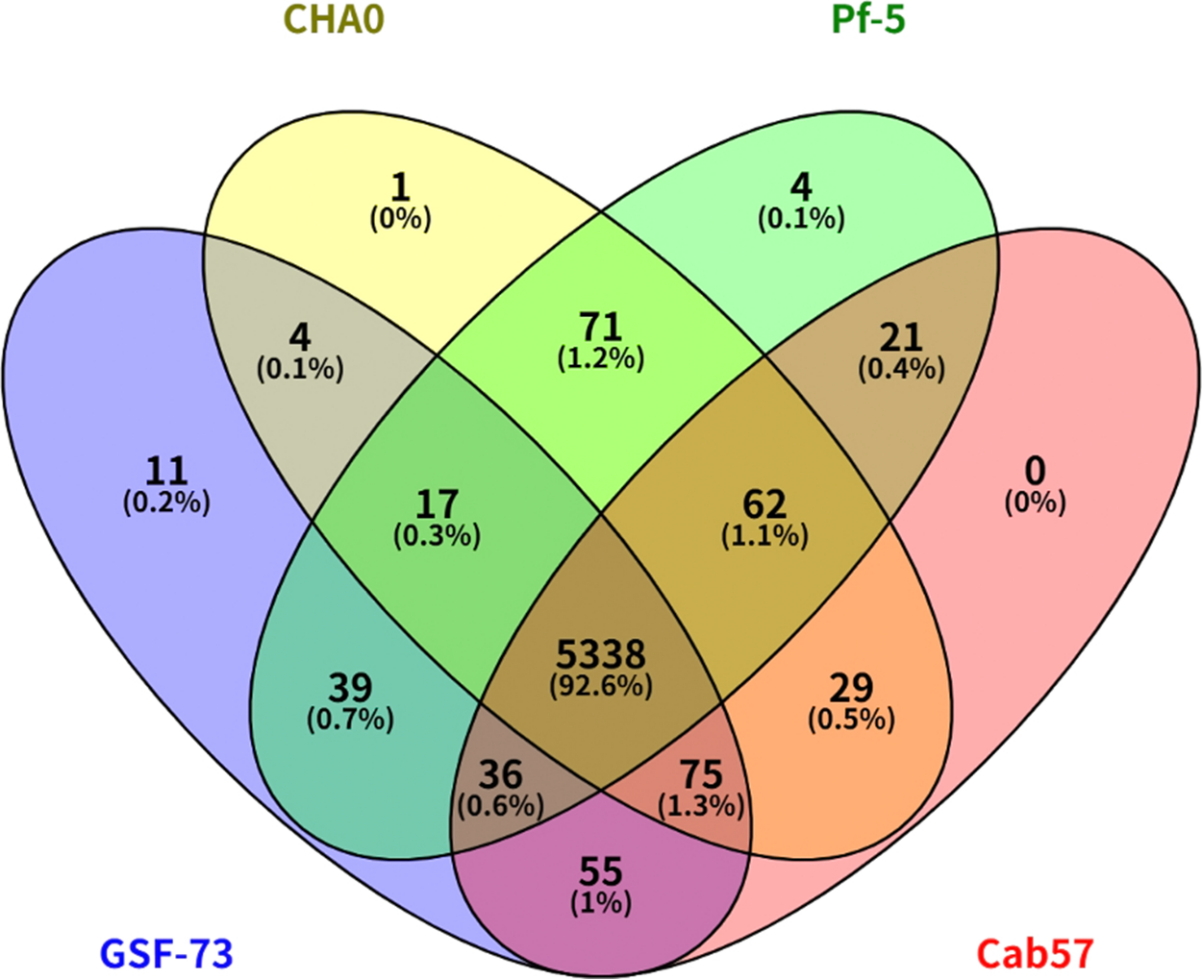
Comparative orthogroup anal­ysis of *Pseudomonas protegens* strains GSF-73, Cab57, CHA0, and Pf-5. The Venn diagram illustrates shared and strain-specific orthogroups identified using OrthoFinder. Numbers indicate orthogroup counts, and percentages represent proportions relative to the total number of orthogroups.

A comparison of COG functional category distributions among *P. protegens* strains revealed largely similar overall patterns across all strains (Fig. S2). However, GSF-73 contained a higher number of assigned genes than the other strains in several major functional categories, including [E] amino acid transport and metabolism, [T] signal transduction mechanisms, [R] general function prediction only, [P] inorganic ion transport and metabolism, and [K] transcription. The homology-based comparative anal­ysis using the RBH method showed that GSF-73 possessed the largest number of strain-specific CDSs among the four strains and shared more CDSs with Cab57 than with CHA0 and Pf-5 (Fig. S3). Consistent with these results, the orthogroup anal­ysis indicated that GSF-73 contained the highest number of strain-specific orthogroups and exhibited greater orthogroup sharing with Cab57 than with CHA0 or Pf-5 (Fig. 2).

A total of 23 gene loci (LOCUS_02480–LOCUS_53310) were identified from the GSF-73–specific orthogroups (Table 5). These loci were predominantly assigned to COG categories related to amino acid transport and metabolism (E), replication, recombination, and repair (L), secondary metabolite biosynthesis, transport, and catabolism (Q), mobilome-associated functions (X), transcription (K), and intracellular trafficking, secretion, and vesicular transport (U). Several genes, including *hisM*, *xerD*, *nfxB*, monoamine oxidase (*aofH*), *fosA5*, and IS1182 family transposases, were detected at multiple loci, indicating the presence of paralogous or closely related gene copies. In addition, GSF-73–specific genes included mobilome-associated elements, such as the integrase/recombinase *fimB* and multiple transposases, components of the type IV secretion system (*virB10* and *trbI*), and secondary metabolism-related genes (*fosA5*, *gloA*, and VOC family proteins), suggesting roles in genome plasticity, intercellular interactions, and specialized metabolic functions.

### *In vitro* antagonistic activity

GSF-73 exhibited strong inhibitory activity against *P. aphanidermatum*, with a mean inhibition rate of 40.2% in dual-culture assays. It also suppressed the growth of *C. orbiculare* and *F. oxysporum* f. sp. *spinaciae*, showing mean inhibition rates of 26.4 and 22.3%, respectively (Table 6). In addition, the strain inhibited *R. pseudosolanacearum*, producing an average inhibition zone diameter of 32.2 mm. Collectively, these results indicate that GSF-73 possesses broad-spectrum antagonistic activity against fungal, oomycete, and bacterial phytopathogens.

### Biocontrol potential against tomato bacterial wilt

**Fig. 3.**
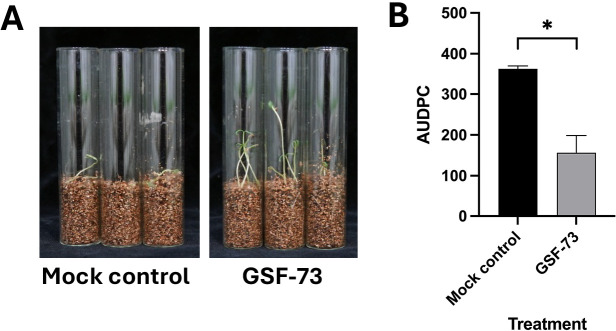
Suppression of tomato bacterial wilt in tomato seedlings by the GSF-73 treatment. (A) Representative images of mock control and GSF-73-treated tomato seedlings in glass tubes 14 days after sowing. (B) AUDPC values 14 days after sowing. Bars represent the mean±standard error from three independent experiments with 15 plants per treatment. An asterisk denotes a significant difference (the Student’s *t*-test, *P*<0.05).

Tomato seedlings in the mock control treatment began to wilt and turn yellow 7–9 days after sowing, and most plants were severely affected or had died by 14 days (Fig. 3A). Average AUDPC values in the mock control reached approximately 363 (Fig. 3B). In contrast, seedlings treated with GSF-73 displayed milder symptoms, and only a few plants exhibited pronounced disease 14 days after sowing (Fig. 3A). As a result, the average AUDPC value in the GSF-73 treatment was significantly reduced to approximately 157 (Fig. 3B).

### Biocontrol potential against *Pythium* root rot in spinach

**Fig. 4.**
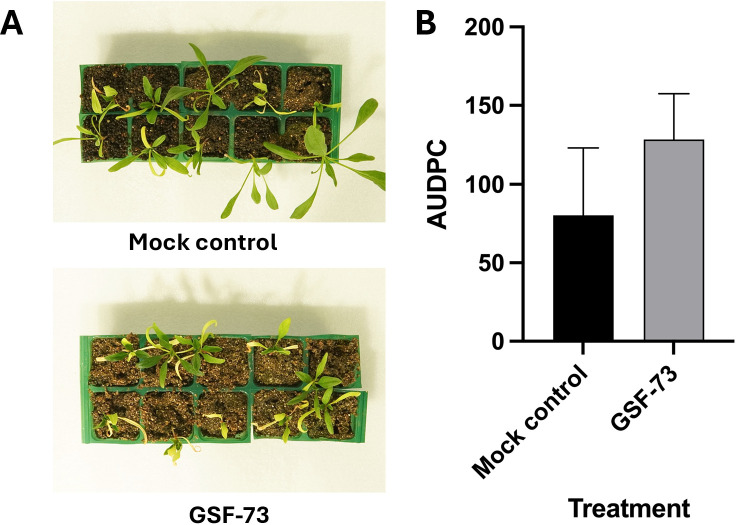
Effects of the GSF-73 treatment on *Pythium*-induced root rot in spinach plants. (A) Representative photographs of spinach seedlings in the mock control and GSF-73–treated groups 14 days after sowing. (B) Area under the disease progress curve (AUDPC) values for the mock control and GSF-73 treatments 14 days after sowing. Bars indicate the mean±standard error from three independent experiments with 10 plants per treatment. According to the Student’s *t*-test, no significant difference was detected between treatments.

In the mock control treatment, wilting or yellowing symptoms began to appear around 9–10 days after sowing. On average, approximately 30% of seedlings had completely wilted, turned yellow, or died by 14 days after sowing (Fig. 4A), and the mean AUDPC value reached 80 (Fig. 4B). In contrast, symptoms emerged earlier in the GSF-73 treatment, appearing around 7–8 days after sowing, and a greater number of seedlings exhibited severe symptoms or mortality by 14 days (Fig. 4A). Consequently, the mean AUDPC value was also higher in the GSF-73 treatment (approximately 128) than in the mock control. However, this difference was not significant (Fig. 4B), suggesting that the GSF-73 treatment promoted, rather than suppressed, *Pythium* root rot in spinach.

### Biocontrol potential against Fusarium wilt in spinach

**Fig. 5.**
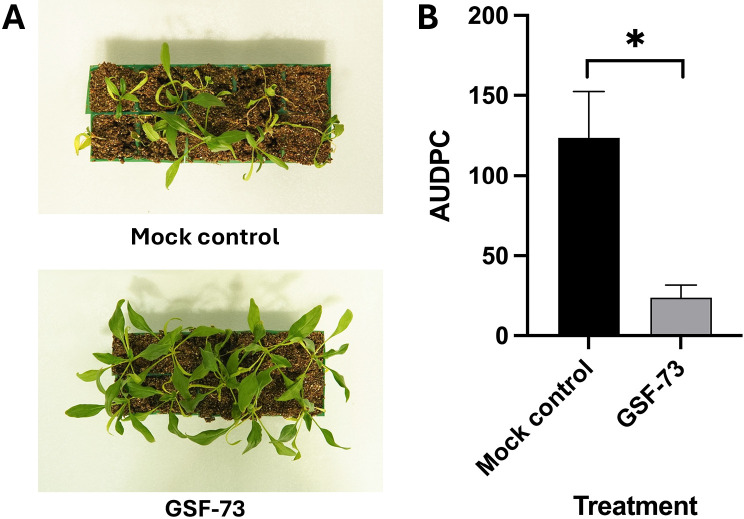
Suppression of Fusarium wilt in spinach seedlings by the GSF-73 treatment. (A) Representative photographs of spinach seedlings in the mock control and GSF-73–treated groups 14 days after sowing. (B) AUDPC values for the mock control and GSF-73 treatments 14 days after sowing. Bars indicate the mean±standard error from three independent experiments with 10 plants per treatment. An asterisk denotes a significant difference (the Student’s *t*-test, *P*<0.05).

In the mock control treatment, *F. oxysporum* f. sp. *spinaciae* severely affected spinach seedlings, and by 14 days after sowing, many had completely wilted and died (Fig. 5A). The average AUDPC value in this treatment reached approximately 124 (Fig. 5B). In contrast, symptoms on seedlings treated with GSF-73 were markedly milder 14 days after sowing (Fig. 5A). As a result, the average AUDPC value was significantly reduced to about 24 (Fig. 5B), representing an 80.7% decrease from the mock control. These results demonstrate the strong biocontrol potential of GSF-73 against Fusarium wilt in spinach.

### Biocontrol potential against anthracnose in cucumber

**Fig. 6.**
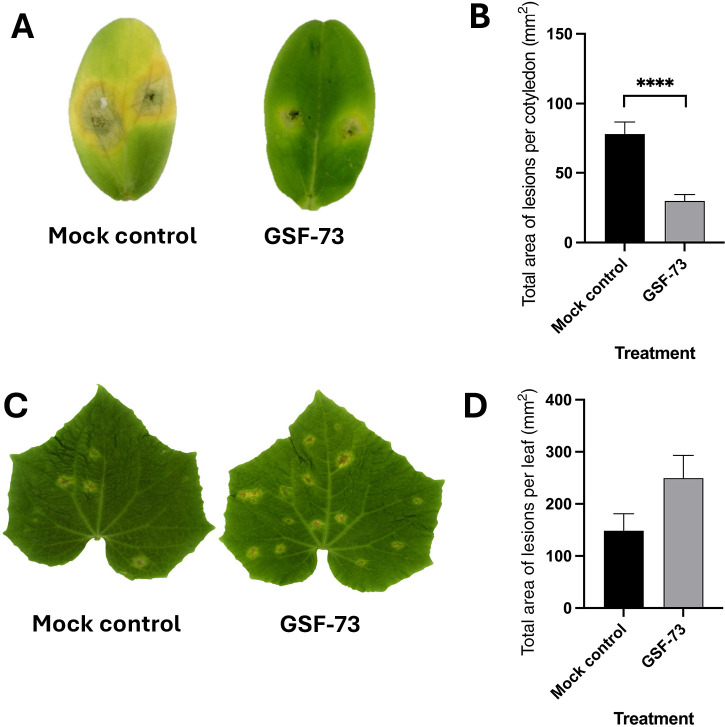
Effects of the GSF-73 treatment on the development of anthracnose symptoms caused by *Colletotrichum orbiculare*. (A) Representative images showing anthracnose symptoms on detached cotyledons in the mock control and GSF-73 treatment groups. (B) Total necrotic lesion area (mm^2^) per cotyledon measured 7 days after the challenge inoculation. Bars represent the mean±standard error from three independent experiments with *n* cotyledons per treatment. **** indicates a significant difference (the Student’s *t*-test, *P*<0.0001). (C) Anthracnose symptoms observed on the leaves of mock control and GSF-73-treated cucumber plants grown in pots. (D) Total necrotic lesion area (mm^2^) on leaves measured 7 days after the pathogen inoculation. Bars show the mean±standard error from three independent experiments with 10 plants per treatment. The Student’s *t*-test indicated no significant difference between treatments.

In the detached cotyledon assay, the GSF-73 treatment strongly inhibited the development of necrotic lesions caused by *C. orbiculare* (Fig. 6A). On average, necrotic lesions in the mock control were approximately 78 mm^2^ per cotyledon, whereas the treatment with GSF-73 significantly reduced lesion sizes to approximately 30 mm^2^ per cotyledon—a 61.9% decrease from the mock control (Fig. 6B).

However, in the pot experiment, GSF-73 did not suppress disease development; instead, it generally enhanced it. Although the difference between treatments was not significant, lesion sizes were consistently larger in the GSF-73 treatment (approximately 249 mm^2^ per leaf) than in the mock control (approximately 148 mm^2^ per leaf) (Fig. 6C and D).

### Biocontrol potential against cucumber downy mildew

**Fig. 7.**
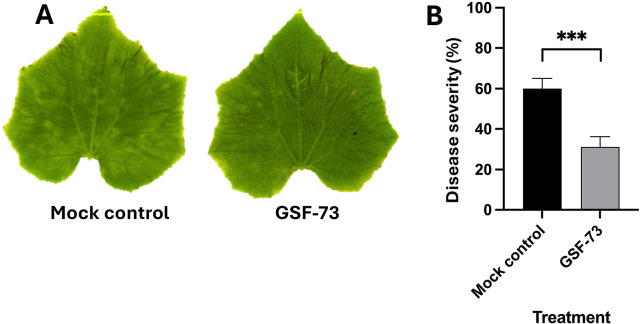
Suppressive effects of GSF-73 on downy mildew symptoms on cucumber leaves. (A) Representative images showing downy mildew symptoms on cucumber leaves 17 days after the inoculation. Numerous pale green to yellow spots appeared on mock control leaves, whereas far fewer were observed on the leaves of cucumber plants treated with GSF-73. (B) Disease severity (%) calculated 17 days after the challenge inoculation. Bars represent the mean±standard error from three independent experiments with five plants per treatment. An asterisk indicates a significant difference (the Student’s *t*-test, *P*<0.05).

In the mock control, downy mildew symptoms were clearly visible on cucumber leaves, with abundant sporulation being detected 17 days after the inoculation (Fig. 7A). Average disease severity in this treatment reached 60% (Fig. 7B). In contrast, leaves treated with GSF-73 showed markedly milder symptoms (Fig. 7A), and average disease severity was significantly reduced to approximately 31% (Fig. 7B), corresponding to a 48% decrease from the mock control.

These results demonstrate that GSF-73 possesses biocontrol potential against cucumber downy mildew.

## Discussion

In the present study, we isolated a strain of *P. protegens*, designated GSF-73, from the rhizosphere of Welsh onion (*A. fistulosum*) collected in Sekigahara, Gifu, Japan, and characterized its genomic features and biocontrol-related phenotypes. The strain exhibited antagonistic activity against multiple plant pathogens and exerted disease-suppressive effects in several plant–pathogen systems, indicating its potential as a biocontrol agent.

The draft genome anal­ysis indicated that the GSF-73 genome spans roughly 7.13‍ ‍Mbp, which is larger than those of the three reference *P. protegens* strains directly compared in this study—Cab57, CHA0, and Pf-5. This larger genome size is accompanied by an increased number of predicted CDSs, suggesting that GSF-73 harbors an expanded genetic repertoire. Nevertheless, the genome size of GSF-73 remains within the upper range reported for *P. protegens*, which typically spans approximately 6.78–7.09 Mb (Zhao *et al.*, 2022; De la Vega-Camarillo *et al.*, 2024). Several previously reported strains exhibit smaller genomes than GSF-73, including H78 (7.03 Mb), FJKB0103 (6.78 Mb), and E1BL2 (7.09 Mb), while the model biocontrol strain Pf-5 has a genome size of approximately 7.07 Mb (Paulsen *et al.*, 2005; Huang *et al.*, 2017; Zhao *et al.*, 2022; De la Vega-Camarillo *et al.*, 2024). These comparisons indicate that, while not exceptional, GSF-73 represents a *P. protegens* strain with a relatively expanded genome.

Genome-wide functional annotation and the COG anal­ysis revealed that GSF-73 exhibits a genomic architecture typical of *P. protegens*, with enrichment in categories related to amino acid transport and metabolism, signal transduction, transcription, and inorganic ion transport. Notably, GSF-73 contained a higher number of genes in these functional categories than the other reference strains, which may reflect enhancements in metabolic flexibility and regulatory capacity rather than unique functional specialization. These traits are considered to be advantageous for rhizosphere competence, allowing rapid adaptation to fluctuating nutrient availability and intense microbial competition (Belda *et al.*, 2016; Li *et al.*, 2021). Orthogroup and RBH-based comparative anal­yses further demonstrated that GSF-73 harbors the largest number of strain-specific CDSs and orthogroups, many of which are associated with secondary metabolism, mobilome elements, transcriptional regulation, and secretion systems. These genes may contribute to strain-specific ecological behaviors and interactions with plants and competing microorganisms.

The genome mining of GSF-73 using antiSMASH identified 19 BGCs associated with the production of secondary metabolites, including eight antimicrobial compounds reported to be produced by *P. protegens*. The corresponding marker genes and/or complete biosynthetic gene sets for all eight antimicrobial BGCs were conserved in GSF-73, indicating that these clusters are likely intact and genetically functional. PLT and DAPG are well-known antibiotics synthesized by *P. protegens* (Haas and Keel, 2003; Gross and Loper, 2009; Loper *et al.*, 2012; Hansen *et al.*, 2022; Dahal *et al.*, 2024). These compounds exhibit strong toxicity toward diverse fungi, oomycetes, and bacteria, including plant-pathogenic species. Consequently, they are considered to be key contributors to the biocontrol capacity of *P. protegens* (Balthazar *et al.*, 2022). Several *P. protegens* strains also produce additional antimicrobial metabolites, such as HCN, pyochelin, pyoverdine, and orfamides (Loper *et al.*, 2012; Huang *et al.*, 2017). The involvement of these compounds, with the exception of orfamides, in the mechanisms by which *Pseudomonas* spp. control plant diseases has also been demonstrated (Buysens *et al.*, 1996; Nandi *et al.*, 2017; Huang *et al.*, 2018; Dobrzyński and Jakubowska, 2025). The model biocontrol strain Pf-5 and the Japanese strain Cab57 were recently reported to produce protegenins, a class of polyene antibiotics with anti-oomycete activity (Murata *et al.*, 2021; Mwanza *et al.*, 2025). GSF-73 harbors the corresponding polyyne BGC, further supporting its genetic capacity to produce a broad spectrum of antimicrobial metabolites targeting diverse phytopathogens.

In addition to these well-characterized antimicrobial BGCs, the genome of GSF-73 contains several uncharacterized BGCs belonging to NRPS, β-lactone, RiPP-like, and CDPS biosynthetic classes. These systems are widespread among bacteria and are known to produce diverse secondary metabolites with antimicrobial, signaling, or ecological functions (Saati-Santamaría *et al.*, 2022; Tamang *et al.*, 2024). Although it was not possible to predict the products encoded by these BGCs based on current antiSMASH annotations, their presence indicates that GSF-73 retains an additional secondary metabolite biosynthetic potential beyond currently characterized compounds in *P. protegens*. However, the biological roles of these uncharacterized clusters remain unknown, and further functional anal­yses will be required to establish whether they contribute to antimicrobial activity or plant-associated fitness.

Moreover, the presence of intact global regulatory genes (*gacS*, *gacA*, *rsmA*, and *uvrY*) in GSF-73 is consistent with the coordinated regulation of antimicrobial production and related traits observed in other *P. protegens* strains. The present study showed that GSF-73 suppressed the growth of *P. aphanidermatum*, *F. oxysporum*, *C. orbiculare*, and *R. pseudosolanacearum* and exhibited biocontrol activity against multiple diseases. In biocontrol assays, the soil application of GSF-73 effectively reduced Fusarium wilt in spinach, caused by *F. oxysporum* f. sp. *spinaciae*, as well as‍ ‍bacterial wilt in tomato seedlings caused by *R. pseudosolanacearum*. These results are consistent with previous findings demonstrating the performance of *P. protegens* strains against soil-borne diseases. Several strains have been documented to control bacterial wilt in tomato and tobacco (Subedi *et al.*, 2020; Wang *et al.*, 2020; Zhao *et al.*, 2024), as well as tomato foot and root rot caused by *F. oxysporum* f. sp. *radicis-lycopersici* (de Weert *et al.*, 2004). Additionally, we observed that dipping cucumber cotyledons in a GSF-73 cell suspension markedly reduced the development of anthracnose symptoms caused by *C. orbiculare*. The application of strains belonging to the genus *Pseudomonas* to aboveground tissues has been reported to effectively suppress anthracnose diseases on chili peppers and banana (Peeran *et al.*, 2014; Sandani *et al.*, 2019). In addition, several investigations have shown the efficacy of the foliar application of *P. protegens* in suppressing gray mold on cherry tomato fruits, cannabis leaves, and grapevine leaves (Andreolli *et al.*, 2019; Balthazar *et al.*, 2022; Ajijah *et al.*, 2023). In this context, the present results suggest that GSF-73 has the potential to suppress anthracnose development when applied to foliar tissues; however, its effectiveness under whole-plant or field conditions remains unclear.

Due to its strong capacity to produce potent antimicrobial metabolites and cell wall–degrading enzymes, research on the biocontrol mechanisms of *P. protegens* has largely focused on direct antimicrobial modes of action. Nevertheless, beyond suppressing pathogen growth and activity, the prevention of infection through the activation of plant immunity—known as induced systemic resistance (ISR)—is also recognized as an important mechanism employed by *P. protegens* (Dobrzyński and Jakubowska, 2025). In the present study, we observed that applying GSF-73 to cucumber roots significantly decreased leaf infection by the downy mildew pathogen *P. cubensis*. These results suggest that GSF-73 protected cucumber plants from downy mildew by triggering ISR. Bahmani *et al.* (2025) recently reported that the soil application of *P. protegens* CHA0 markedly reduced the severity of downy mildew in grapevines caused by *Plasmopara viticola*. They further showed that a treatment with CHA0 enhanced several key defense-related responses, including increases in defense enzyme activity, phenolic compound accumulation, hydrogen peroxide levels, and the expression of pathogen-responsive genes, such as *LOX9*, *PR4*, and *OSM1*. Since most of the up-regulated genes were responsive to jasmonate (JA), they proposed that the CHA0-mediated suppression of downy mildew in grapevines primarily occurred through the enhancement of systemic defenses via the JA signaling pathway. Although defense-related responses were not analyzed in the present study, these findings are consistent with the possibility that GSF-73 enhances systemic disease resistance. However, the involvement of specific defense signaling pathways, such as JA– or salicylic acid (SA)–dependent pathways, has yet to be exami­ned.

Conversely, we also identified unexpected negative effects of GSF-73 in the biocontrol assays. Spinach seedlings treated with GSF-73 consistently showed more severe *Pythium* root rot caused by *P. aphanidermatum* than mock-treated plants. This outcome was unexpected because GSF-73 harbors BGCs for the production of anti-oomycete antibiotics, including DAPG and PLT. Moreover, GSF-73 exhibited strong inhibitory activity against *P. aphanidermatum* in dual-culture assays; therefore, we anticipated that it may mitigate disease severity caused by this pathogen. However, these results contrast with previous findings showing that *P. protegens* strains effectively suppressed damping-off caused by *P. ultimum* (Kraus and Loper, 1992; Maurhofer *et al.*, 1994; Takeuchi *et al.*, 2014). Even more unexpectedly, the treatment of cucumber seeds and roots with GSF-73 generally increased anthracnose severity on leaves. This contradicts the results of the detached cotyledon assay, in which dipping cotyledons in the GSF-73 suspension significantly reduced anthracnose development. At present, the mechanisms underlying these contrasting effects remain unclear. One potential explanation is that GSF-73 strongly stimulated the JA signaling pathway in spinach and cucumber, which may have inadvertently increased susceptibility to *Pythium* root rot and anthracnose. The JA- and SA–dependent pathways typically antagonize one another; therefore, heightened resistance to pathogens sensitive to JA-mediated defenses often correlates with increased vulnerability to pathogens reliant on SA-mediated defenses, and *vice versa* (Grant and Lamb, 2006). Chen *et al.* (1999) also reported the importance of SA-mediated defenses in systemic resistance against *P. aphanidermatum* induced by a biocontrol *Pseudomonas* strain. Liu *et al.* (2008) showed that the JA treatment increased the susceptibility of cucumber plants to *C. orbiculare*. A recent study by Beskrovnaya *et al.* (2020) further supports this hypothesis and provides additional insights. They demonstrated that two plant growth-promoting *Pseudomonas* strains elicited induced systemic susceptibility (ISS) to *P. syringae* pv. tomato DC3000 in *Arabidopsis* by suppressing specific SA-dependent responses rather than activating ISR. Instead, *Arabidopsis* plants treated with these strains developed enhanced JA-dependent resistance against herbivores. The authors identified a distinct genomic locus containing several genes required for triggering ISS. Notably, this ISS locus also played an important role in rhizosphere colonization by these *Pseudomonas* strains. Therefore, GSF-73 may likewise harbor a similar locus that promotes its rhizosphere colonization during root introduction, but simultaneously elicits ISS by reducing SA-mediated defenses against certain pathogens. Although the present study did not assess defense signaling or examine the presence or function of an ISS-associated locus in GSF-73, a similar mechanism cannot be excluded. Further studies will be required to clarify whether GSF-73 modulates plant immunity through JA–, SA–, or other signaling pathways and how this modulation relates to both disease suppression and enhancement.

In the present study, we isolated and characterized a rhizosphere-derived strain of *P. protegens*, GSF-73, which exhibited disease-suppressive activity against multiple plant diseases. The genome anal­ysis revealed that GSF-73 possesses a relatively large genome and harbors a repertoire of BGCs and regulatory genes characteristic of biocontrol-associated *P. protegens* strains. At the same time, a treatment with GSF-73 enhanced disease severity in specific host–pathogen combinations, underscoring the complexity and context dependency of plant–microbe interactions. A comprehensive evaluation of both beneficial and adverse effects will be essential before practical applications, and further studies are needed to elucidate the molecular bases underlying the contrasting effects of GSF-73.

## Acknowledgements

This work was supported by JSPS KAKENHI (grant 22H02344) from the Ministry of Education, Culture, Sports, Science and Technology of Japan.

### Compliance with ethical standards

*Conflict of Interest*: All authors declare that there is no conflict of interest.

## Citation

Yuliana, R., Ikeda, S., Ikagawa, Y., Yamauchi, K., Kuwahara, K., Baharom, N. A., et al. (2026) Isolation and Genomic Characterization of a Novel Japanese Strain of *Pseudomonas protegens* and an Evaluation of Its Biocontrol Potential. *Microbes Environ ***41**: ME25086.

https://doi.org/10.1264/jsme2.ME25086

## Supplementary Material

Supplementary Material

## Figures and Tables

**Table 1.** General genomic features of *Pseudomonas protegens* strains GSF-73, Cab57, CHA0, and Pf-5.

Strain	Genome size (bp)	CDS no.	G+C content (%)	rRNA genes no.	tRNA genes no.	Accession no.
GSF-73	7,132,059	6,705	63	16	74	AP045182
Cab57	6,827,892	6,178	63.3	16	75	NC004129
CHA0	6,867,980	6,188	63.4	15	72	AP014522
Pf-5	7,074,893	6,344	63.3	16	75	CP003190

**Table 2.** Average nucleotide identity (ANI) and TETRA value comparison between strain GSF-73 and genome sequences of its closest related strains

Other *Pseudomonas protegens* strains	Cab57	CHA0	Pf-5
Average Nucleotide Identity to GSF-73	98.26	98.14	97.81
TETRA value to GSF-73	0.99983	0.99975	0.99971

**Table 3.** Predicted biosynthetic gene clusters in the genome of *Pseudomonas protegens* strain GSF-73

Region	BGC type	Most similar known cluster	Similarity confidence
1	Polyyne	Protegencin	High
2	Arylpolyene	APE Vf	Low
3	RiPP-like	—	—
4	CDPS	—	—
5	Homoserine lactone	—	—
6	NRPS, phosphonate	Orfamide A/C	High
7	Hydrogen cyanide	Hydrogen cyanide	High
8	T1PKS, RiPP-like	Pyoluteorin	High
9	NRP-metallophore, NRPS	Enantio-pyochelin	High
10	Other	Pyrrolnitrin	High
11	Betalactone	—	—
12	NRP-metallophore, NRPS, ranthipeptide	Pyoverdine (Pf-5)	Low
13	NRPS	Pyoverdine (Pf-5)	Low
14	NAGGN	—	—
15	NRPS	—	—
16	Terpene-precursor	—	—
17	Redox-cofactor	—	—
18	T3PKS	2,4-diacetylphloroglucinol	High
19	RiPP-like	—	—

“—” indicates that no confidently similar biosynthetic gene cluster was identified in the antiSMASH database. Similarity confidence was assigned by antiSMASH v8.0.4 based on the percentage of homologous genes shared between query and reference clusters.

**Table 4.** Secondary metabolite–related genes and biosynthetic gene clusters identified in *Pseudomonas protegens* strain GSF-73.

Function	Representative genes/cluster (markers)	Presence in GSF-73	Detected markers	Missing markers
Protegencin (polyyne)	antiSMASH region001 (polyyne BGC)	Detected	antiSMASH region001 (polyyne)	—
Orfamide A/Orfamide C (CLP)	*ofaA*+*ofaB*+ofaC (antiSMASH region006; NRPS)	Detected	*ofaA*, *ofaB*, *ofaC*	—
Hydrogen cyanide (HCN)	*hcnA*+*hcnB*+*hcnC*	Detected	*hcnA*, *hcnB*, *hcnC*	—
Pyoluteorin (PLT)	*pltM*+*pltR*+*pltA*+*pltB*+*pltC*+*pltD*+*pltE*+*pltF*+*pltG*+*pltH*+*pltI*+*pltJ*+*pltK*+*pltN* (transport)	Detected	*pltM* (K14257-like), *pltR*, *pltA*, *pltB*, *pltC*, *pltD* (K14257-like), *pltE*, *pltF*, *pltG*, *pltH*, *pltI*, *pltJ*, *pltK*, *pltN*	—
Enantio-pyochelin (siderophore)	*pchA*+*pchB*+*pchC*+*pchE*+*pchF* (antiSMASH region009)	Detected	*pchA*, *pchB*, *pchC*, *pchF*, *pchE*, *pchR*	—
Pyrrolnitrin (PRN)	*prnA*+*prnB*+*prnC*+*prnD*	Detected	*ctcP*, *cts4*, *prnC*, *prnA*, *rebH*, *ktzQ*, *prnB*, *prnD*	—
Pyoverdine (siderophore)	*pvdA*+*pvdE*+*fpvA* (pyoverdine-related)	Detected	*pvdQ*, *quiP*, TC.FEV.OM1, *fhuE*, *fpvA*, *fptA*, *pvdA*, *SIDA*, *pvdE*, *pac*	—
2,4-Diacetylphloroglucinol (2,4-DAPG)	*phlA*+*phlB*+*phlC*+*phlD*	Detected	*phlG*, *phlA*, *phlC*, *phlB*, *phlD*	—
Gac/Rsm global regulation	*gacS*+*gacA*+*rsmA/csrA*	Detected	*uvrY*, *gacA*, *varA*, *barA*, *gacS*, *varS*, *csrA*, *ksgA*	—
Rhizoxin	*rzxA–rzxF*+*rzxG-rzxI*	Not detected	—	*rzxA–rzxF* + *rzxG-rzxI*

Detection was based on genome annotation and an antiSMASH anal­ysis. Detected and missing marker genes are shown for each functional category. *Putative *pltD* and *pltM* genes were inferred based on KO annotation and conserved gene synteny within the pyoluteorin biosynthetic gene cluster (region008) predicted by antiSMASH.

**Table 5.** Genes uniquely detected in *Pseudomonas protegens* strain GSF-73 based on a comparative ortholog anal­ysis.

LOCUS	KO no.	COG no.	COG Category	Gene	Product Name
LOCUS_02480		COG0765	Amino acid transport and metabolism (E)	HisM	ABC-type amino acid transport system, permease component
LOCUS_04460		COG0765	Amino acid transport and metabolism (E)	HisM	ABC-type amino acid transport system, permease component
LOCUS_32350	K00274	COG1231	Amino acid transport and metabolism (E)	MAO, *aofH*	Monoamine oxidase
LOCUS_32390	K00274	COG1231	Amino acid transport and metabolism (E)	MAO, *aofH*	Monoamine oxidase
LOCUS_03490		COG4974	Replication, recombination and repair (L)	XerD	Site-specific recombinase XerD
LOCUS_08910		COG4974	Replication, recombination and repair (L)	XerD	Site-specific recombinase XerD
LOCUS_20740		COG4974	Replication, recombination and repair (L)	XerD	Site-specific recombinase XerD
LOCUS_25370	K21265	COG0346	Secondary metabolites biosynthesis, transport, and catabolism (Q)	*fosA5*	Glutathione S-transferase fosA5
LOCUS_34930		COG0346	Secondary metabolite biosynthesis, transport, and catabolism (Q)	GloA	Catechol 2,3-dioxygenase or related enzymes, vicinal oxygen chelate (VOC) family
LOCUS_39080	K21265	COG0346	Secondary metabolites biosynthesis, transport, and catabolism (Q)	*fosA5*	Glutathione S-transferase fosA5
LOCUS_25380		COG3666	Mobilome: prophages, transposons (X)	—	Transposase, IS1182 family
LOCUS_39070		COG3666	Mobilome: prophages, transposons (X)	—	Transposase, IS1182 family
LOCUS_32160	K18294	COG1309	Transcription (K)	*nfxB*	TetR/AcrR family transcriptional regulator, mexCD-oprJ operon repressor
LOCUS_32210	K18294	COG1309	Transcription (K)	*nfxB*	*TetR/AcrR* family transcriptional regulator, mexCD-oprJ operon repressor
LOCUS_36150		COG2948	Intracellular trafficking, secretion, and vesicular transport (U)	VirB10	Type IV secretory pathway, *VirB10* component
LOCUS_53310	K20533	COG2948	Intracellular trafficking, secretion, and vesicular transport (U)	*trbI*	Type IV secretion system protein TrbI
LOCUS_15680		COG0582	Replication, recombination and repair, Mobilome: prophages, transposons (L, X)	*FimB*	Integrase/recombinase, includes phage integrase
LOCUS_08880					Hypothetical protein
LOCUS_34810					VOC family protein
LOCUS_36450					Hypothetical protein
LOCUS_53090					Hypothetical protein
LOCUS_53180					Hypothetical protein
LOCUS_53260					Hypothetical protein

* KO and COG annotations were assigned using an integrated comparative ortholog anal­ysis. Multiple loci for the same gene indicate paralogous or closely related copies.

**Table 6.** *In vitro* antagonistic activity of *Pseudomonas protegens* strain GSF-73 against fungal, oomycete, and bacterial plant pathogens

Pathogens	Growth inhibition (%)	Diameter of halo (mm)
*Pythium aphanidermatum*	22.9±4.7	—
*Fusarium oxysporum* f. sp. *spinaciae*	22.3±2.4	—
*Colletotrichum orbiculare*	26.4±5.5	—
*Ralstonia pseudosolanacearum*	—	32.2±13.0

Antagonistic activity against fungal and oomycete pathogens was assessed using the dual-culture method, whereas activity against *R. pseudosolanacearum* was evaluated using the spot-on-the-lawn method. Values represent the mean±standard deviation from three independent experiments.
