# Supplementary material for: Isolation and Genomic Characterization of a Novel Japanese Strain of Pseudomonas protegens and an Evaluation of Its Biocontrol Potential
Source: Microbes Environ. 2026 May 29;41(2):ME25086. doi: 10.1264/jsme2.ME25086 (PMC13293703; doi:10.1264/jsme2.ME25086)
Supplement: Supplementary file 1 — Supplementary Material [file 41_25086_s1.pdf]

Supplementary Table S1. Bacterial isolates selected through fluorescent colony screening and identified by 16S rRNA gene sequencing

Strains	Source	Closest species	Similarity (%)	Accession No.	Location
GTS-1	Soil	*Pseudomonas farris*	99.71	GCA_019145235.1_21	Tottori Prefecture
GTS-3	Soil	*Pseudomonas jessenii*	99.77	NIWT01000013	Tottori Prefecture
GTS-4	Soil	*Pseudomonas vancouverensis*	99.65	AJ011507	Tottori Prefecture
GTS-6.1	Soil	*Pseudomonas vancouverensis*	99.81	AJ011507	Tottori Prefecture
GTS-6.2	Soil	*Pseudomonas vancouverensis*	99.81	AJ011507	Tottori Prefecture
GTS-6.3	Soil	*Pseudomonas vancouverensis*	99.81	AJ011507	Tottori Prefecture
GTS-6.4	Soil	*Pseudomonas vancouverensis*	99.81	AJ011507	Tottori Prefecture
GTS-6.5	Soil	*Pseudomonas vancouverensis*	99.81	AJ011507	Tottori Prefecture
GTS-6.6	Soil	*Pseudomonas vancouverensis*	99.81	AJ011507	Tottori Prefecture
GTS-6.7	Soil	*Pseudomonas vancouverensis*	99.81	AJ011507	Tottori Prefecture
GTS-16	Soil	*Burkholderia stagnalis*	99.92	LK023502	Tottori Prefecture
GTS-17	Soil	*Bacillus altitudinis*	100	ASJC01000029	Tottori Prefecture
GTS-18	Soil	*Burkholderia stagnalis*	99.92	LK023502	Tottori Prefecture
GTS-19	Soil	*Pseudomonas vancouverensis*	99.77	AJ011507	Tottori Prefecture
GTS-21	Soil	*Enterobacter ludwigii*	99.78	JTLO01000001	Tottori Prefecture
GTS-23	Soil	*Pseudomonas vancouverensis*	99.71	AJ011507	Tottori Prefecture
GGF-1	Soil	*Serratia marcescens*	99.93	JMPQ01000005	Gifu University Experimental Field Plot
GGF-5	Soil	*Stenotrophomonas maltophilia*	99.78	JALV01000036	Gifu University Experimental Field Plot
GGF-6	Soil	*Pseudoxanthomonas mexicana*	99.92	AF273082	Gifu University Experimental Field Plot
GGF-7	Soil	*Pseudoxanthomonas mexicana*	100	AF273082	Gifu University Experimental Field Plot
GGF-11	Soil	*Cellulosimicrobium funkei*	99.83	AY501364	Gifu University Experimental Field Plot
GGF-13	Soil	*Serratia marcescens*	99.92	JMPQ01000005	Gifu University Little Forest

GGF-14	Soil	*Dyella marensis*	99.93	AM939778	Gifu University (North Farm)
GGF-17	Soil	*Pseudomonas azerbaijanorientalis*	99.68	GCA_019139795.1_1	Gifu University (North Farm)
GGF-20	*Verbena brasiliensis* (Brazilian vervain)	*Pseudomonas mosselii*	99.93	AF072688	Gifu University
GGF-21	*Verbena brasiliensis* (Brazilian vervain)	*Pseudomonas mosselii*	99.85	AF072688	Gifu University
GGF-24	*Verbena brasiliensis* (Brazilian vervain)	*Pseudomonas plecoglossicida*	100	BBIV01000080	Gifu University
GGF-25	*Verbena brasiliensis* (Brazilian vervain)	*Pseudomonas asiatica*	100	MH517510	Gifu University
GGF-26	*Verbena brasiliensis* (Brazilian vervain)	*Pseudomonas guariconensis*	100	FMYX01000029	Gifu University
GGF-27	*Verbena brasiliensis* (Brazilian vervain)	*Pseudomonas plecoglossicida*	100	BBIV01000080	Gifu University
GGF-28	*Verbena brasiliensis* (Brazilian vervain)	*Pseudomonas plecoglossicida*	100	BBIV01000080	Gifu University
GGF-29	*Verbena brasiliensis* (Brazilian vervain)	*Bacillus cereus*	100	AE016877	Gifu University
GGF-32	*Desmodium paniculatum* (Panicled Tick-Trefoil)	*Bacillus toyonensis*	100	CP006863	Gifu University
GGF-35	*Desmodium paniculatum* (Panicled Tick-Trefoil)	*Pseudomonas bijieensis*	99.64	MT835388	Gifu University
GGF-36	*Amaranthus viridis* (Slender amaranth)	*Pseudomonas tohonis*	99.93	LC645211	Gifu University
GGF-38	*Conyza canadensis* (Canadian horseweed)	*Pseudomonas asiatica*	100	MH517510	Gifu University
GGF-39	*Persicaria longiseta* (Creeping Smartweed)	*Pseudomonas japonica*	99.63	BBIR01000146	Gifu University
GGF-41	*Conyza canadensis* (Canadian horseweed)	*Pseudomonas tohonis*	99.92	LC645211	Gifu University

GGF-43	*Allium macrostemon* (Japanese wild onion)	*Pseudomonas ceruminis*	99.85	MT498800	Gifu University
GGF-44	*Allium macrostemon* (Japanese wild onion)	*Pseudomonas kribbensis*	100	CP029608	Gifu University
GGF-45	*Allium macrostemon* (Japanese wild onion)	*Pseudomonas sichuanensis*	100	QKVM01000121	Gifu University
GGF-46	*Allium macrostemon* (Japanese wild onion)	*Pseudomonas fitomaticsae*	100	MZ773500	Gifu University
GGF-47	*Allium macrostemon* (Japanese wild onion)	*Pseudomonas vlassakiae*	100	JABWRP010000055	Gifu University
GGF-48	*Allium macrostemon* (Japanese wild onion)	*Paenarthrobacter nicotinovorans*	100	X80743	Gifu University
GGF-49	*Allium macrostemon* (Japanese wild onion)	*Pseudomonas fitomaticsae*	100	MZ773500	Gifu University
GGF-50	*Allium macrostemon* (Japanese wild onion)	*Pseudomonas kurunegalensis*	99.91	AM911650	Gifu University
GGF-51	*Allium macrostemon* (Japanese wild onion)	*Pseudomonas triticicola*	99.78	GCA_019145375.1_2	Gifu University
GGF-53	*Allium macrostemon* (Japanese wild onion)	*Pseudomonas kurunegalensis*	100	AM911650	Gifu University
GGF-55	*Allium macrostemon* (Japanese wild onion)	*Pseudomonas kribbensis*	100	CP029608	Gifu University
GGF-56	*Allium macrostemon* (Japanese wild onion)	*Pseudomonas vlassakiae*	100	JABWRP010000055	Gifu University
GGF-57	*Allium macrostemon* (Japanese wild onion)	*Pseudomonas vlassakiae*	100	JABWRP010000055	Gifu University
GGF-58	*Allium macrostemon* (Japanese wild onion)	*Pseudomonas alloputida*	100	LT718459	Gifu University
GSF-1	*Allium fistulosum* (Welsh onion)	*Pseudomonas kurunegalensis*	99.91	AM911650	Sekigahara, Gifu Prefecture
GSF-2	*Allium fistulosum* (Welsh onion)	*Pseudomonas putida*	99.82	AP013070	Sekigahara, Gifu Prefecture
GSF-6	Soil	*Chromobacterium piscinae*	99.11	AJ871127	Sekigahara, Gifu Prefecture

GSF-7	Soil	*Xanthomonas maliensis*	99.61	KF992843	Sekigahara, Gifu Prefecture
GSF-9	Soil	*Lysobacter enzymogenes*	100	jgi.1095734	Sekigahara, Gifu Prefecture
GSF-15	Soil	*Sphingobium yanoikuyae*	99.92	JH992904	Sekigahara, Gifu Prefecture
GSF-16	Soil	*Agromyces allii*	99.59	DQ673873	Sekigahara, Gifu Prefecture
GSF-19	*Cucurbita pepo* (pumpkin)	*Pseudomonas fulva*	100	BBIQ01000036	Sekigahara, Gifu Prefecture
GSF-22	*Cucurbita pepo* (pumpkin)	*Pseudomonas promysalinigenes*	99.93	JABWRQ010000026	Sekigahara, Gifu Prefecture
GSF-23	*Cucurbita pepo* (pumpkin)	*Pseudomonas fulva*	100	BBIQ01000036	Sekigahara, Gifu Prefecture
GSF-24	*Cucurbita pepo* (pumpkin)	*Pseudomonas fulva*	98.77	BBIQ01000036	Sekigahara, Gifu Prefecture
GSF-25	*Perilla frutescens* var. *crispa* (Perilla)	*Roseateles chitinivorans*	99.71	MH077556	Sekigahara, Gifu Prefecture
GSF-27	*Perilla frutescens* var. *crispa* (Perilla)	*Lysobacter enzymogenes*	99.75	jgi.1095734	Sekigahara, Gifu Prefecture
GSF-29	*Allium tuberosum* (Chinese chives)	*Kosakonia cowanii*	99.61	BBEU01000098	Sekigahara, Gifu Prefecture
GSF-30	*Allium tuberosum* (Chinese chives)	*Stenotrophomonas maltophilia*	99.86	JALV01000036	Sekigahara, Gifu Prefecture
GSF-31	*Allium tuberosum* (Chinese chives)	*Pseudomonas inefficax*	100	OPYN01000008	Sekigahara, Gifu Prefecture
GSF-32	*Allium tuberosum* (Chinese chives)	*Pseudomonas alvandae*	99.93	GCA_019141525.1_1	Sekigahara, Gifu Prefecture
GSF-33	*Allium tuberosum* (Chinese chives)	*Paenarthrobacter nicotinovorans*	99.83	X80743	Sekigahara, Gifu Prefecture
GSF-34	*Allium tuberosum* (Chinese chives)	*Burkholderia sola*	100	GCA_029268985.1_m	Sekigahara, Gifu Prefecture
GSF-36	*Allium tuberosum* (Chinese chives)	*Burkholderia sola*	100	GCA_029268985.1_m	Sekigahara, Gifu Prefecture
GSF-39	*Allium fistulosum* (Welsh onion)	*Kosakonia cowanii*	99.83	BBEU01000098	Sekigahara, Gifu Prefecture

GSF-40	*Allium fistulosum* (Welsh onion)	*Comamonas koreensis*	98.95	AF275377	Sekigahara, Gifu Prefecture
GSF-41	*Allium fistulosum* (Welsh onion)	*Burkholderia aenigmatica*	99.91	LR760817	Sekigahara, Gifu Prefecture
GSF-42	*Allium fistulosum* (Welsh onion)	*Comamonas koreensis*	98.94	AF275377	Sekigahara, Gifu Prefecture
GSF-44	*Allium fistulosum* (Welsh onion)	*Serratia marcescens*	99.77	JMPQ01000005	Sekigahara, Gifu Prefecture
GSF-45	*Allium fistulosum* (Welsh onion)	*Pseudomonas alloputida*	99.93	LT718459	Sekigahara, Gifu Prefecture
GSF-46	*Allium fistulosum* (Welsh onion)	*Pseudomonas rhodesiae*	99.93	AF064459	Sekigahara, Gifu Prefecture
GSF-49	*Celosia argentea* (Plumed cockscomb)	*Roseateles chitinivorans*	99.41	MH077556	Sekigahara, Gifu Prefecture
GSF-50	*Celosia argentea* (Plumed cockscomb)	*Roseateles chitinivorans*	99.41	MH077556	Sekigahara, Gifu Prefecture
GSF-55	*Celosia argentea* (Plumed cockscomb)	*Pseudomonas solani*	100	LC744517	Sekigahara, Gifu Prefecture
GSF-56	*Celosia argentea* (Plumed cockscomb)	*Bacillus cereus*	100	AE016877	Sekigahara, Gifu Prefecture
GSF-57	*Cucumis sativus L.* (Cucumber)	*Pseudomonas fakonensis*	100	GCA_019139895.1_1	Sekigahara, Gifu Prefecture
GSF-58	*Cucumis sativus L.* (Cucumber)	*Pseudomonas hamedanensis*	99.7	JABWQT010000081	Sekigahara, Gifu Prefecture
GSF-59	*Cucumis sativus L.* (Cucumber)	*Pseudomonas arcuscaelestis*	99.66	OD946146	Sekigahara, Gifu Prefecture
GSF-60	*Cucumis sativus L.* (Cucumber)	*Pseudomonas maumuensis*	99.56	GCA_019139675.1_1	Sekigahara, Gifu Prefecture
GSF-61	*Allium fistulosum* (Welsh onion)	*Pseudomonas paraglycinae*	100	BQHR01000050	Sekigahara, Gifu Prefecture
GSF-62	*Allium fistulosum* (Welsh onion)	*Pseudomonas paraglycinae*	100	BQHR01000050	Sekigahara, Gifu Prefecture

GSF-63	*Allium tuberosum* (Chinese chives)	*Pseudomonas inefficax*	100	OPYN01000008	Sekigahara, Gifu Prefecture
GSF-67	*Allium tuberosum* (Chinese chives)	*Pseudomonas guariconensis*	99.85	FMYX01000029	Sekigahara, Gifu Prefecture
GSF-68	*Allium tuberosum* (Chinese chives)	*Pseudomonas glycinae*	100	MG692779	Sekigahara, Gifu Prefecture
GSF-69	*Allium fistulosum* (Welsh onion)	*Pseudomonas koreensis*	99.92	AF468452	Sekigahara, Gifu Prefecture
GSF-70	*Allium fistulosum* (Welsh onion)	*Pseudomonas kurunegalensis*	99.92	AM911650	Sekigahara, Gifu Prefecture
GSF-73	*Allium fistulosum* (Welsh onion)	*Pseudomonas protegens*	100	CP003190	Sekigahara, Gifu Prefecture
GSF-74	*Allium fistulosum* (Welsh onion)	*Pseudomonas kurunegalensis*	99.92	AM911650	Sekigahara, Gifu Prefecture
GSF-75	*Persicaria capitata* (Smart weed)	*Pseudomonas mosselii*	99.93	AF072688	Sekigahara, Gifu Prefecture
GSF-78	*Capsicum annuum L.* (Green pepper)	*Pseudomonas sichuanensis*	100	QKVM01000121	Sekigahara, Gifu Prefecture
GSF-79	*Melissa officinalis* (Lemon balm)	*Pseudomonas putida*	99.85	AP013070	Sekigahara, Gifu Prefecture
GSF-80	*Lycopersicon esculentum* (Cherry tomatoes)	*Pseudomonas kurunegalensis*	100	AM911650	Sekigahara, Gifu Prefecture
GSF-81	*Lycopersicon esculentum* (Cherry tomatoes)	*Pseudomonas kribbensis*	100	CP029608	Sekigahara, Gifu Prefecture
GSF-82	*Lycopersicon esculentum* (Cherry tomatoes)	*Pseudomonas japonica*	99.82	BBIR01000146	Sekigahara, Gifu Prefecture
GSF-83	*Lycopersicon esculentum* (Cherry tomatoes)	*Pseudomonas mosselii*	99.91	AF072688	Sekigahara, Gifu Prefecture

GSF-84	*Lycopersicon esculentum* (Cherry tomatoes)	*Pseudomonas allokribbensis*	100	CP062252	Sekigahara, Gifu Prefecture
GSF-86	*Salvia splendens* (Scarlet sage)	*Pseudomonas putida*	99.85	AP013070	Sekigahara, Gifu Prefecture
GSF-88	*Salvia splendens* (Scarlet sage)	*Pseudomonas umsongensis*	99.92	NIWU01000003	Sekigahara, Gifu Prefecture
GSF-89	*Capsicum annuum* L. (Green pepper)	*Pseudomonas otitidis*	99.91	AY953147	Sekigahara, Gifu Prefecture
GSF-90	*Capsicum annuum* L. (Green pepper)	*Pseudomonas otitidis*	99.92	AY953147	Sekigahara, Gifu Prefecture
GSF-91	*Capsicum annuum* L. (Green pepper)	*Pseudomonas otitidis*	99.91	AY953147	Sekigahara, Gifu Prefecture
GSF-92	*Capsicum annuum* L. (Green pepper)	*Pseudomonas otitidis*	99.91	AY953147	Sekigahara, Gifu Prefecture
GSF-93	*Capsicum annuum* L. (Green pepper)	*Pseudomonas otitidis*	99.91	AY953147	Sekigahara, Gifu Prefecture
GSF-96	*Cucumis sativus L.* (Cucumber)	*Pseudomonas glycinae*	99.91	MG692779	Sekigahara, Gifu Prefecture
GSF-97	*Allium fistulosum* (Welsh onion)	*Pseudomonas aeruginosa*	100	BAMA01000316	Sekigahara, Gifu Prefecture
GSF-99	*Allium fistulosum* (Welsh onion)	*Pseudomonas juntendi*	99.82	MK680061	Sekigahara, Gifu Prefecture
GSF-100	*Allium fistulosum* (Welsh onion)	*Pseudomonas paraglycinae*	100	BQHR01000050	Sekigahara, Gifu Prefecture
GSF-101	*Melissa officinalis* (Lemon balm)	*Pseudomonas aeruginosa*	100	BAMA01000316	Sekigahara, Gifu Prefecture
GSF-103	*Persicaria capitata* (Smart weed)	*Pseudomonas tohonis*	99.91	LC645211	Sekigahara, Gifu Prefecture
GSF-105	*Lycopersicon esculentum* (Cherry tomatoes)	*Pseudomonas glycinae*	99.91	MG692779	Sekigahara, Gifu Prefecture

GSF-106	*Lycopersicon esculentum* (Cherry tomatoes)	*Pseudomonas kribbensis*	100	CP029608	Sekigahara, Gifu Prefecture
GSF-107	*Lycopersicon esculentum* (Cherry tomatoes)	*Pseudomonas paraglycinae*	100	BQHR01000050	Sekigahara, Gifu Prefecture

Supplementary Table S2. Detailed antiSMASH predictions of biosynthetic gene clusters in the genome of *Pseudomonas protegens* strain GSF-73

Region	Type	From	To	Most similar known cluster	Similarity confidence*
Region 1	Polyyne	300,056	327,381	Protegencin	High
Region 2	Arylpolyene	523,065	566,682	APE Vf	Low
Region 3	RiPP-like	1,517,235	1,528,125	None	None
Region 4	CDPS	1,617,566	1,638,315	None	None
Region 5	Hserlactone	2,116,519	2,137,085	None	None
Region 6	NRPS, phosphonate	2,437,561	2,511,212	Orfamide A/orfamide C	High
Region 7	Hydrogen cyanide	2,947,456	2,960,425	Hydrogen cyanide	High
Region 8	T1PKS, RiPP-like	3,159,814	3,212,562	Pyoluteorin	High
Region 9	NRP-metallophore, NRPS	4,235,587	4,286,712	Enantio-pyochelin	High
Region 10	Other	4,367,082	4,408,167	Pyrrolnitrin	High
Region 11	beta-lactone	4,722,765	4,745,979	None	None
Region 12	NRP-metallophore, NRPS, ranthipeptide	4,860,969	4,964,887	Pf-5 pyoverdine	Low
Region 13	NRPS	5,017,332	5,070,348	Pf-5 pyoverdine	Low
Region 14	NAGGN	5,215,834	5,230,665	None	None
Region 15	NRPS	5,535,035	5,577,908	None	None
Region 16	Terpene-precursor	6,341,108	6,361,995	None	None
Region 17	Redox-cofactor	6,516,097	6,538,262	None	None
Region 18	T3PKS	6,807,227	6,848,276	2,4-diacetylphloroglucinol	High
Region 19	RiPP-like	7,078,584	7,089,429	None	None

*Similarity confidence refers to the proportion of homologous genes shared between the query and matched clusters, as defined by antiSMASH v8.0.4. antiSMASH categorizes this confidence into three levels: "high" (similarity ≥75%), "medium" (similarity ≥50% to <75%), and "low" (similarity ≥15% to <50%). Clusters labeled as "None" showed no confident similarity to any known BGCs in the antiSMASH database.

Fig. S1.

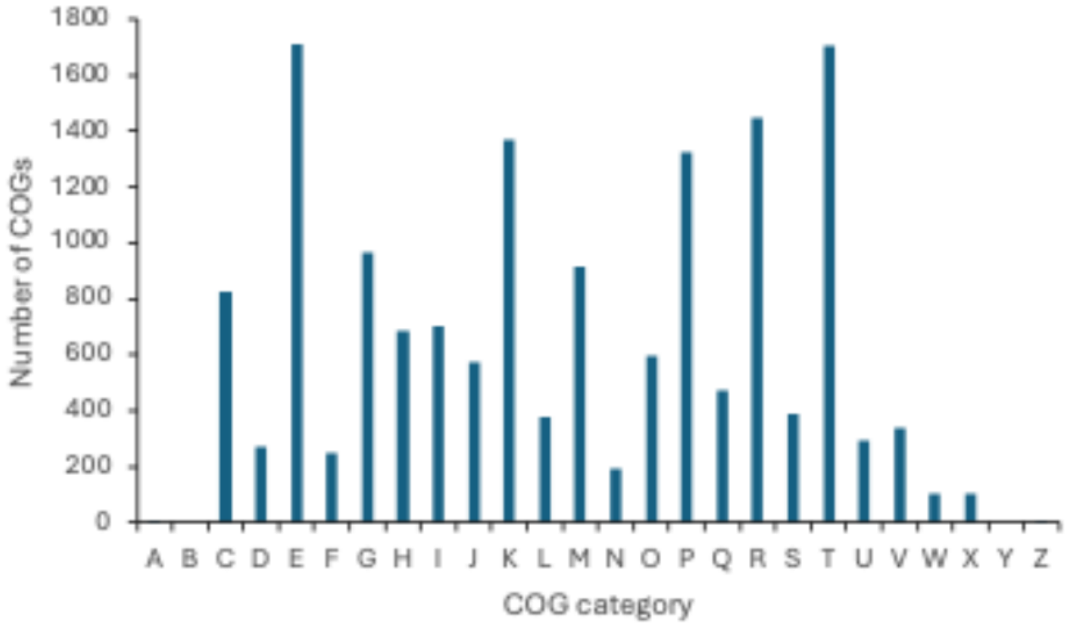

Fig. S1. Distribution of Clusters of Orthologous Groups (COG) functional categories in *Pseudomonas protegens* strain GSF-73. Bars indicate the number of genes assigned to each COG category (A–Z) based on COG annotation of predicted protein sequences. A, RNA processing and modification; B, chromatin structure and dynamics; C, energy production and conversion; D, cell cycle control and mitosis; E, amino acid transport and metabolism; F, nucleotide transport and metabolism; G, carbohydrate transport and metabolism; H, coenzyme transport and metabolism; I, lipid transport and metabolism; J, translation and ribosomal structure; K, transcription; L, replication, recombination and repair; M, cell wall/membrane/envelope biogenesis; N, cell motility; O, post-translational modification, protein turnover and chaperones; P, inorganic ion transport and metabolism; Q, secondary metabolite biosynthesis, transport and catabolism; R, general function prediction only; S, function unknown; T, signal transduction mechanisms; U, intracellular trafficking and secretion; V, defense mechanisms; W, extracellular structures; X, mobilome (prophages and transposons); Y, nuclear structure; and Z, cytoskeleton.

Fig. S2.

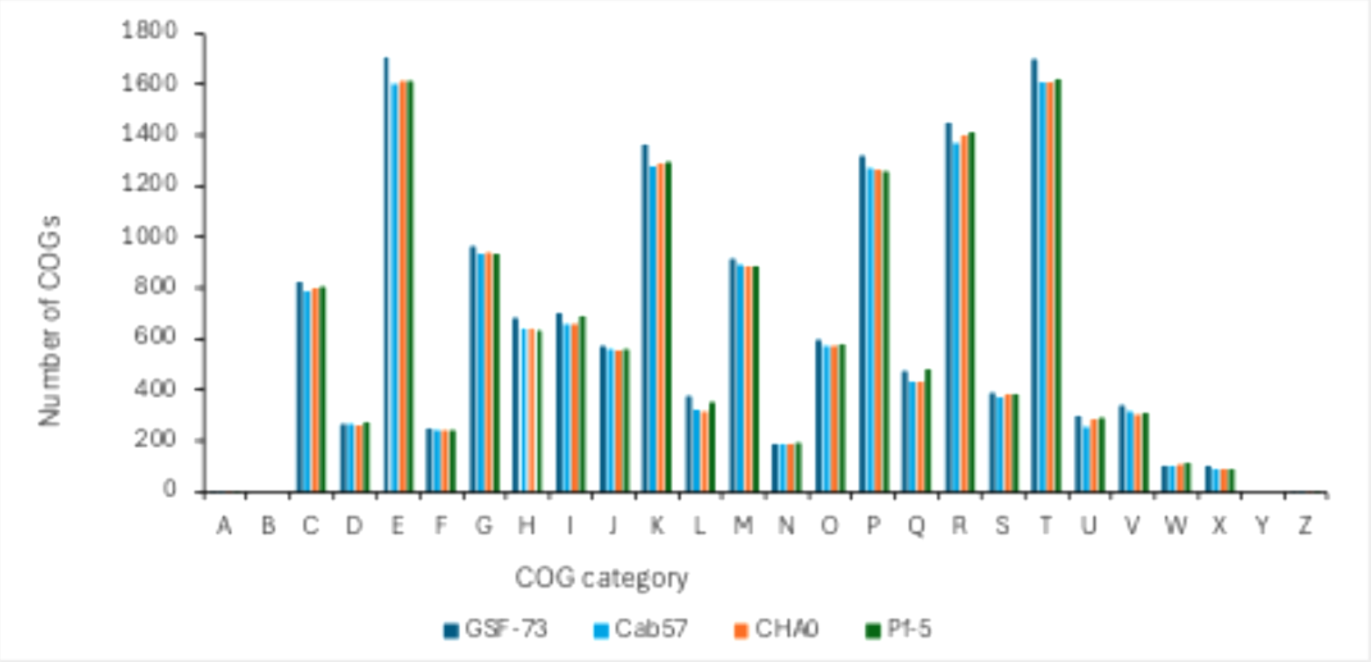

Fig S2. The bar chart shows the number of predicted coding sequences (CDSs) assigned to each Cluster of Orthologous Groups (COG) category from A to Z: A, RNA processing and modification; B, chromatin structure and dynamics; C, energy production and conversion; D, cell cycle control and mitosis; E, amino acid transport and metabolism; F, nucleotide transport and metabolism; G, carbohydrate transport and metabolism; H, coenzyme transport and metabolism; I, lipid transport and metabolism; J, translation and ribosomal structure; K, transcription; L, replication, recombination and repair; M, cell wall/membrane/envelope biogenesis; N, cell motility; O, post-translational modification, protein turnover and chaperones; P, inorganic ion transport and metabolism; Q, secondary metabolite biosynthesis, transport and catabolism; R, general function prediction only; S, function unknown; T, signal transduction mechanisms; U, intracellular trafficking and secretion; V, defense mechanisms; W, extracellular structures; X, mobilome (prophages and transposons); Y, nuclear structure; and Z, cytoskeleton.

Fig. S3.

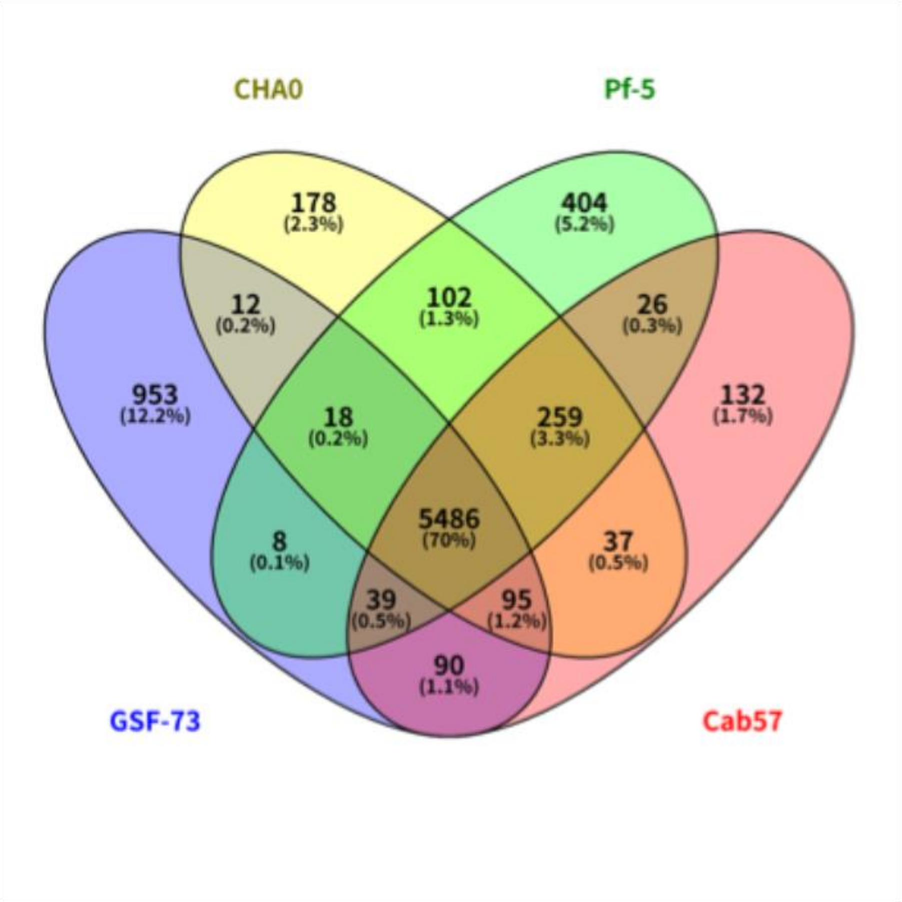

Fig. S3. Venn diagram comparing CDSs of *Pseudomonas protegens* strains GSF-73, Cab57, CHA0, and Pf-5 based on homology-based comparative analysis
